# Clinical and Molecular Characteristics Associated with Survival in Advanced Melanoma Treated with Checkpoint Inhibitors

**DOI:** 10.1155/2018/6279871

**Published:** 2018-07-17

**Authors:** Sunil Badami, Sunil Upadhaya, Ravi Kanth Velagapudi, Pushyami Mikkilineni, Ranju Kunwor, Samer Al Hadidi, Ghassan Bachuwa

**Affiliations:** ^1^Hurley Medical Center/Michigan State University, 1 Hurley Plaza, Flint, Michigan 48503, USA; ^2^Mercy St. Vincent Medical Center, 2213 Franklin Ave, Toledo, OH 43604, USA; ^3^MacNeal Health Center, 3249 South Oak Park Ave. Berwyn, Illinois 60402, USA; ^4^Baylor College of Medicine, One Baylor Plaza-BCM 187, Houston, TX 77030, USA

## Abstract

**Background:**

We performed meta-analysis to gather more evidence regarding clinical-molecular subgroups associated with better overall survival (OS) in advanced melanoma treated with checkpoint inhibitors.

**Materials and Methods:**

We performed a systematic search of PubMed, Scopus, Cochrane Library, and clinical trial.gov. Randomized clinical trials that compared a checkpoint inhibitor (nivolumab or pembrolizumab) with investigator choice chemotherapy or ipilimumab were included in our study. Hazard ratios (HR) and confidence interval (CI) were calculated for progression-free survival (PFS) and OS for each subgroup using generic inverse model along with the random effect method.

**Results:**

A total of 6 clinical trials were eligible for the meta-analysis. OS was prolonged in wild BRAF subgroup (HR 0.65, 95% CI 0.49-0.85, p 0.002), Programmed cell death subgroup (PD-1+) (HR 0.57, 95% CI 0.41-0.80, p 0.001), and high lactate dehydrogenase (LDH) level subgroup (HR 0.60, 95% CI 0.38-0.95, p 0.03). Similarly, we found increased OS in eastern cooperative oncology group (ECOG) 1, males and age >65 years subgroups.

**Conclusions:**

Checkpoint inhibitors significantly increased OS in patients with wild BRAF, positive PD-1, and high LDH. However, results should be interpreted keeping in mind associated significant heterogeneity. The results of this study should help in designing future clinical trials.

## 1. Introduction

Advanced melanoma (regionally metastatic melanoma stage III) and metastatic disease (stage IV) has been the deadliest form of cutaneous malignancy. According to the latest statistics from the Surveillance, Epidemiology, and End Results (SEER) 18 registry, the incidence of melanoma in the United States continues to rise. A total of 87,110 cases were reported in 2017. Although there is an uptrend of new cases, the 5-year survival rate has been trending upward, with the latest being 19.9% [1].

In 2011, a new era began in management of advanced melanoma with United States Food and Drug Administration (FDA) approval of anti-CTLA-4 (cytotoxic T lymphocyte antigen-4) targeted therapy (ipilimumab) [2], which gave promising results, such as better overall survival (OS), response rate, and progression-free survival (PFS).

Other molecular targets were also encouraging, including targeting of B-Rapidly Accelerated Fibrosarcoma (BRAF) gene V600 mutation in 2011[3] (vemurafenib, dabrafenib) and mitogen-activated kinase (MEK) pathway inhibitors (trametinib) approved in 2013[4].

The latest addition to immunotherapy are anti-programed cell death agents (PD-1), which target the programmed cell death pathway and its ligands. Tumors escape the host immune system by evading checkpoints of T cells and natural killer cells. To date, the most effective immune checkpoint inhibitor is developed against PD-1 and its ligand (PD-L1) [5]. It is also noted that the expression of PD-L1, which is also associated with melanoma, is higher in tumors with poor prognosis [6, 7].

The anti-PD-1 agent and monoclonal antibody pembrolizumab got an accelerated approval by the FDA based on the phase 1 study KeyNote (KN) 001 in 2014[8]. It was initially approved for disease progressed on ipilimumab/anti-BRAF treatment, but subsequent studies CheckMate (CM) 067, CM 069 (nivolumab), and KN 002 (pembrolizumab) [9, 10] proved the superiority of checkpoint inhibitors. As of now, National Comprehensive Cancer Network (NCCN) guidelines recommend these agents either for first-line monotherapy or in combination with CTLA-4 inhibitor.

However, there is not much evidence in terms of which subgroup of patients with advanced melanoma treated with checkpoint inhibitors have better survival outcomes. Available data regarding survival benefit of checkpoint inhibitors in patients based on BRAF status and PD1 expression are contradictory. Results from KN 002 trial and CM 037 trial have shown trend towards better survival in wild BRAF and PD1+ subgroup of patients compared to BRAF mutated and PD1 negative subgroups, respectively, in patients treated with checkpoint inhibitors. However, KN 006 trial, CM 066 trial, and CM 067 trial did not show any survival difference based on BRAF status and PD1 expression [8, 9, 11–13].

As checkpoint inhibitors stimulate immune response of the patient against tumor antigens, response to these drugs is affected by clinical and molecular profile of the patient. Gender, age, and functional status affect immune response [14–16]. Serum lactate dehydrogenase (LDH) is an important staging marker and elevated level is associated with higher tumor burden with worse survival outcomes. Similar to BRAF status and PD1 expression, there is conflicting evidence regarding survival benefit based on LDH level in patient treated with checkpoint inhibitors [8, 9, 11, 12].

We conducted this meta-analysis and systematic review to gather more evidence regarding survival in different clinical-molecular subgroups based on PD-1 gene expression status, BRAF gene mutation status, serum LDH level, and demographic factors such as age, sex, and ECOG (eastern cooperative oncology group) functional status.

## 2. Materials and Methods

**Figure 1:**
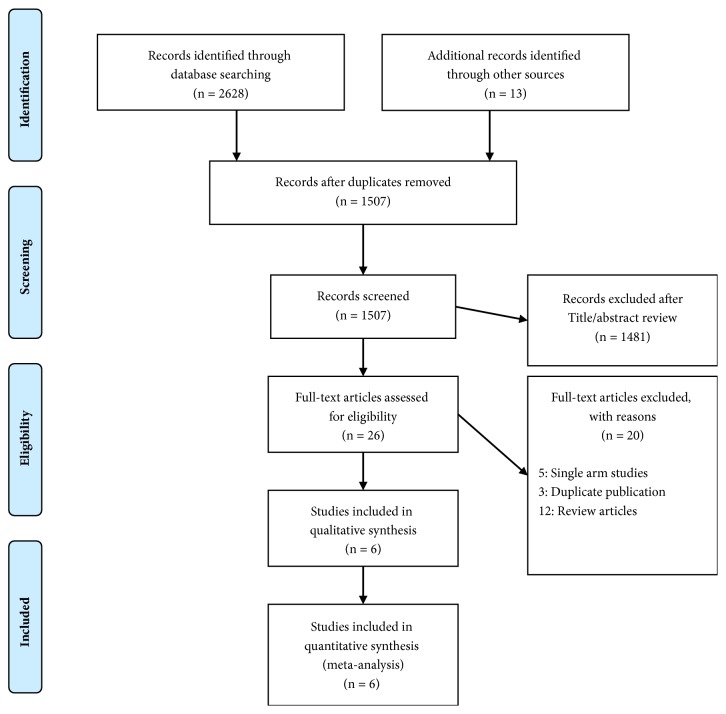
PRISMA (Preferred Reporting Items for Systematic Reviews and Meta-Analyses) diagram of the review.

We used the Preferred Reporting Items for Systematic Reviews and Meta-Analyses (PRISMA) model for our analysis (Figure 1) [17]. We performed a systematic search of PubMed, Scopus, Cochrane Library, and clinicaltrial.gov from inception of database till June 2018. Articles published only in English language were considered. We used the following terms in two groups for the search: Group 1: “metastatic melanoma” and “advanced melanoma”; Group 2: “anti-programmed cell death receptor 1 monoclonal antibody”, “anti-PD-1 monoclonal antibody”, “PD-1”, “pembrolizumab” and “nivolumab”. We used the all-field strategy (search all text). We used the Boolean operator “OR” for searches using words in the same group. We combined the search results from both groups using the Boolean operator “AND” to narrow down the search. We searched the bibliographies of the retrieved publications to get additional relevant studies. Elaborate search strategy is provided in Supplementary Table 1. For the initial search, we went through the title/abstract of the articles. After the initial search, a full text review of the selected articles was done. We only used published data for the analysis. In addition, we looked for relevant articles in the bibliographies of the selected articles. Initial search resulted in 2641 citations. After exclusion of duplicate citations, abstracts of remaining 1507 articles were reviewed by 2 authors (P.M. and R.K.). Out of 1507 articles 1481 were excluded based on review of abstract and title. Final full text review of remaining 26 articles was done by 3 authors separately (S.B., S.U., and R.V.) and, in case of any conflict during the review, final decision was done by two senior authors (G.B. and S.H.) panel. In case of search yielding more than one paper in the same study population, we chose the most recently published article with outcome relevant to our study.

Inclusion criteria for our analysis followed the PICOS model: population, patients with advanced melanoma; intervention, anti-PD immunotherapy; comparison, anti-PD immunotherapy versus investigator choice chemotherapy or anti CTLA-4 immunotherapy; outcome: clinical outcomes based on clinical and genetic stratification; and study design, randomized studies only [18].

Based on the inclusion criteria, only 6 randomized studies (5, phase III; 1, phase II) were included in the analysis. Primary outcome and baseline characteristics were extracted by 3 authors (P.M., R.K., and S.B.) (Tables 1, 2, and 3). In case of any confusion, final decision was made by panel of senior authors (G.B. and S.H.). The final Delphi (Table 4) list of included articles was extracted by two authors (S.B. and S.U.). In addition, studies with potentially high risk of bias are listed in Supplementary Table 2.

We used RevMan 5.3 for windows (Cochrane Collaboration, Oxford, United Kingdom) for the analysis. Random models and hazard ratios (HRs) were used to assess the outcomes. PFS and OS were calculated using the generic inverse model along with the random model method. A two-tailed p value of less than 0.05 was taken as significant. Heterogeneity of <30% was considered low, 30%-60% was considered moderate, and >60% was considered substantial.

We conducted meta-regression using Comprehensive Meta-analysis version 3 (Biostat Inc, Englewood, USA). We used categorical (sample size greater or less than 500 and control arm using ipilimumab or not) and continuous (age, gender, ECOG status 0, BRAF mutation, and PD 1 status, year of publication) potential moderator for estimating the effect on heterogeneity and effect size using univariate regression on overall survival rates.

We used funnel plot to assess for publication bias. Funnel plot for this review is symmetrical, so risk of publication bias is low (Supplementary Figure 1).

## 3. Results and Discussions

### 3.1. Results

#### 3.1.1. Overall Survival

**Figure 2:**
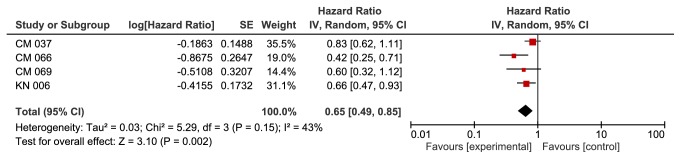
**Forest plot for overall survival wild BRAF subgroup. **CI: confidence interval; CM: CheckMate; KN: KeyNote; IV: inverse variance; SE: standard error.

**Figure 3:**
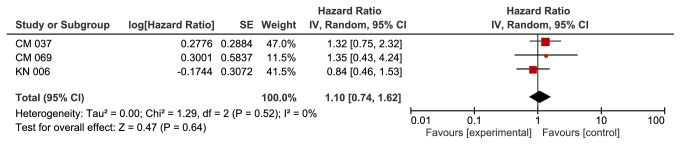
**Forest plot for overall survival mutated BRAF subgroup. **CI: confidence interval; CM: CheckMate; KN: KeyNote; IV: inverse variance; SE: standard error.

**Based on BRAF mutation group**, we found a significant improvement in the anti-PD-1 group in patients with wild BRAF gene (HR 0.65, 95% CI 0.49-0.85, p value of 0.002). This outcome was associated with moderate heterogeneity of 43% using I^2^ (Figure 2). We did not find a significant difference between the two groups in patients with BRAF mutation (HR 1.10, 95% CI 0.74-1.62, p value of 0.64). Heterogeneity was 0% for this outcome (Figure 3).

**Figure 4:**
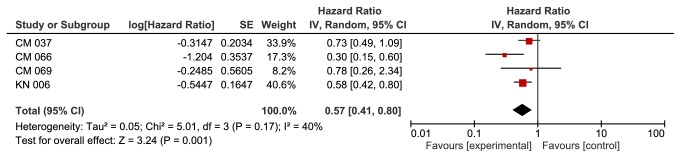
**Forest plot for overall survival PD1 positive subgroup. **CI: confidence interval; CM: CheckMate; KN: KeyNote; IV: inverse variance; SE: standard error.

**Based on PD-1 status**, we found a significant improvement in the anti-PD-1 group in patients with PD-1+ (HR 0.57, 95% CI 0.41-0.80, p value of 0.001). This outcome was associated with moderate heterogeneity of 40% using I^2^ (Figure 4). We did not find a significant difference between the two groups in patients with PD-1- (HR 0.80, 95% CI 0.51-1.27, p value of 0.35).

**Figure 5:**
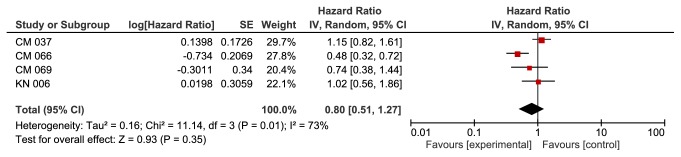
**Forest plot for overall survival PD1 negative subgroup. **CI: confidence interval; CM: CheckMate; KN: Keynote; IV: inverse variance; SE: standard error.

Heterogeneity was 73% for this outcome (Figure 5).

**Based on ECOG**, we found a significant improvement in the anti-PD-1 group in patients with an ECOG of 1 (HR 0.70, 95% CI 0.55-0.90, p value of 0.004). This outcome was associated with very low heterogeneity of 0%. We did not find a significant difference between the two groups in patients with an ECOG of 0 (HR 0.66, 95% CI 0.41-1.05, p value of 0.08). Heterogeneity was 78% for this outcome.

**Based on gender**, we found a significant improvement in the anti-PD-1 group in males (HR 0.60, 95% CI 0.40-0.91, p value of 0.02). This outcome was associated with substantial heterogeneity of 73%. We did not find a significant difference between the two groups in females (HR 0.81, 95% CI 0.61-1.07, p value of 0.13). Heterogeneity was 14% for this outcome.

**Based on age**, we found a significant improvement in the anti-PD-1 group in patients age 65 or older (HR 0.61, 95% CI 0.42-0.88, p value of 0.008). This outcome was associated with moderate heterogeneity of 41%. We did not find a significant difference between the two groups in patients younger than 65 (HR 0.75, 95% CI 0.50-1.11, p value of 0.15). Heterogeneity was 68% for this outcome.

**Figure 6:**
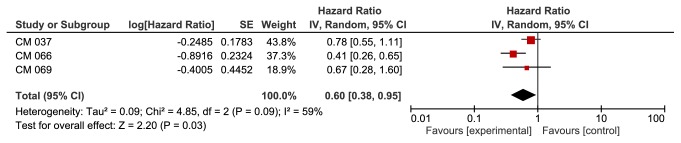
**Overall survival in high lactate dehydrogenase (LDH) subgroup. **CI: confidence interval; CM: CheckMate; KN: KeyNote; IV: inverse variance; SE: standard error.

**Figure 7:**
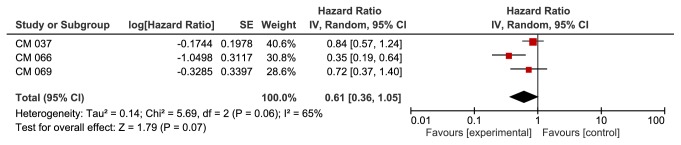
**Overall survival in normal lactate dehydrogenase (LDH) subgroup. **CI: confidence interval; CM: CheckMate; KN: KeyNote; IV: inverse variance; SE: standard error.

**Based on lactate dehydrogenase (LDH) levels**, we found a significant improvement in the anti-PD-1 group in patients with high LDH levels (HR 0.60, 95% CI 0.38-0.95, p value of 0.03). This outcome was associated with moderate heterogeneity of 59% (Figure 6). We did not find a significant difference between the two groups in patients with normal LDH levels (HR 0.61, 95% CI 0.36-1.05, p value of 0.07). Heterogeneity was 65% for this outcome (Figure 7).

#### 3.1.2. Progression-Free Survival

**Based on BRAF mutation group**, we found a significant improvement in the anti-PD-1 group in patients with wild BRAF gene (HR 0.49, 95% CI 0.43-0.55, p value of <0.00001). This outcome was associated with very low heterogeneity of 0% using I^2^. We did not find a significant difference between the two groups in patients with BRAF mutation (HR 0.73, 95% CI 0.52-1.04, p value of 0.08). Heterogeneity was 26% for this outcome.

**Based on PD-1 status**, we found a significant improvement in the anti-PD-1 group in patients with PD-1+ (HR 0.52, 95% CI 0.40-0.67, p value of <0.00001). This outcome was associated with very low heterogeneity of 0% using I^2^. We did not find a significant difference between the two groups in patients with PD-1- (HR 0.48, 95% CI 0.19-1.20, p value of 0.12). Heterogeneity was 83% for this outcome.

**Based on ECOG**, we found a significant improvement in the anti-PD-1 group in patients with an ECOG of 1 (HR 0.51, 95% CI 0.37-0.69, p value of <0.0001). This outcome was associated with moderate heterogeneity of 53%. We found a significant difference favoring the anti-PD-1 group in patients with an ECOG of 0 (HR 0.5 3, 95% CI 0.41-0.68, p value of <0.00001). Heterogeneity was 0% for this outcome.

**Based on gender**, we found a significant improvement in the anti-PD-1 groups in males (HR 0.51, 95% CI 0.42-0.63, p value of <0.00001) and females (HR 0.50, 95% CI 0.36-0.70, p value of <0.0001). This outcome was associated with heterogeneity of 0% and 36% in males and females, respectively.

**Based on age**, we found a significant improvement in the anti-PD-1 groups in patients age 65 or older (HR 0.56, 95% CI 0.44-0.70, p value of <0.00001) and patients younger than 65 (HR 0.46, 95% CI 0.33-0.65, p value of <0.0001). This outcome was associated with heterogeneity of 0% and 57% in patients age 65 or older and patients younger than 65, respectively.

**Based on lactate dehydrogenase (LDH) levels**, We found a significant improvement in the anti-PD-1 groups in patients with high LDH levels (HR 0.59, 95% CI 0.42-0.83, p value of 0.003) and normal LDH levels (HR 0.41, 95% CI 0.31-0.53, p value of <0.00001). This outcome was associated with very low heterogeneity of 0% in both groups.

**Heterogeneity:** we did the leave-one-out analysis to find out the cause of high heterogeneity in all outcomes. The cause of high heterogeneity in the outcomes is due to results of CM 066 study. This study only included patients with wild BRAF, so has results strongly favoring PD1 inhibitor compared to other treatment arm.

We also conducted meta-regression to find the cause of heterogeneity. We did not find any impact of age (p value 0.26), gender (p value of 0.28), PD status (p value of 0.68), year of publication (p value of 0.42), sample size (p value of 0.65), and control arm (p value of 0.58) on heterogeneity. However, ECOG status and LDH levels were associated with decrease in heterogeneity from 46.05 % to 41.41% (p value of 0.28) (Supplementary Figure 2) and 27.29 % (p value of 0.1212) (Supplementary Figure 3), respectively; however, both moderators did not reach level of statistical significance. BRAF mutation status was associated with statistically significant decrease in heterogeneity from 46.05 % to 0.00% (p value of 0.0088) (Supplementary Figure 4).

## 4. Discussion

To the best of our knowledge, this is the first meta-analysis to compare outcomes based on clinical and molecular characteristics of PD-1 antibody use in patients with advanced melanoma. We did not find any significant improvement in OS in patients with BRAF mutation. Similarly, we did not find any improvement in PFS in patients with BRAF mutation. However, both OS and PFS were significantly better in patients with wild-type BRAF.

To further stratify the results, we did sensitivity analysis, turn by turn leaving out studies using combination therapy of PD-1/CTLA-4 inhibitor and studies using nivolumab. As expected, there was no change in the significance of statistical outcomes in OS and PFS in either of the groups. NCCN guidelines does not have clear recommendation regarding the use of PD-1 inhibitors in advanced melanoma. The NCCN guidelines recommend using BRAF inhibitor in BRAF mutation patients if early response is deemed necessary.

One possible explanation for these results could be due to aggressive nature of melanoma with BRAF mutation compared to wild variant [19]. As of now, evidence for choice of agent in first-line therapy in BRAF mutated patients is indirectly based on retrospective reviews and meta-analysis. However, there is an ongoing clinical trial comparing BRAF/MEK versus checkpoint inhibitors, which hold the key for more concrete evidence.

NCT02224781 is a randomized phase III trial with initial ipilimumab/nivolumab followed by dabrafenib/trametinib at progression versus the reverse approach and the primary outcome for the study is the OS at 2 years of follow-up. Another trial (NCT02631447) is being conducted in Europe with three treatment groups: initial BRAF/MEK (LGX818/MEK162) followed by ipilimumab/nivolumab at progression, the reverse approach, and the third cohort with 8 weeks of BRAF/MEK with a forced switch to combination immunotherapy, and then BRAF/MEK at progression. These trials are designed to be head-to-head comparisons of efficacy of BRAF/MEK versus CTLA-4/PD-1 therapy; the results will be available no later than 2021 and 2022, respectively.

The PFS was significantly better irrespective of LDH levels in our study compared to investigators choice chemotherapy/ipilimumab. However, we did not find any significant survival benefit in the normal LDH population. We found a significant increase in OS in the PD-1 group with high LDH. In this outcome, a total of only 3 studies were included. After the removal of CM 066[12], which included patients with only wild BRAF, whereas the other 2 studies included patients with wild and mutated BRAF, the statistical significance of the OS in high LDH patients was lost. In the same CM 066 [12] study, prespecified subgroup analysis OS was significantly improved irrespective of LDH levels. Interestingly, in 2 published studies (one with 78 patients and the other with 617 patients) with mutated BRAF, OS was better in patients with normal LDH than those with high LDH when a BRAF/MEK inhibitor was used [20, 21]. The association of baseline elevated LDH and poor prognosis is known but utility of LDH as a marker of disease activity and ability to detect occult metastases has been so far ineffective [22]. Although there are three retrospective reviews conducted by Long et al. [19, 23, 24] which report association of baseline LDH and serial monitoring of LDH as an effective marker for targeted therapy, the main drawback remains lack of PDL1 status information, which correlates with better prognosis compared to BRAF mutated patients, as reported in randomized trials and included and confirmed by our results [9, 11]. In conclusion, evidence regarding association between LDH level and survival outcomes for patients treated with checkpoint inhibitors remains unclear, which needs to be further clarified with future trials with possible monitoring of serum LDH during targeted therapy. As we discussed initially, we did not find a significant benefit in patients with mutated BRAF with the use of a PD-1 inhibitor.

Additionally, there is evidence from a well-sized randomized trial (CM 066) that there was no correlation between LDH levels and OS [12]. With respect to PD-1 status in our analysis, we found a significant improvement in both OS and PFS in PD-1+ patients. However, the cutoff point for positivity of PD-1 expression in the included studies was not uniform. CM 037, 066, and 069 [12, 25] used 5% as the cutoff for positivity, whereas KN 006 used 1% as the cutoff. On sensitivity analysis of OS in patients with positive PD-1 using the leave-one-out method, removal of the only KN006 led to a loss of statistical significance. In CM 037 and CM 069, OS was not statistically significant, irrespective of PD-1 status. However, in CM 066 OS was significant in both groups (PD-1 + and PD-1 -). One possible hypothesis for these overall results could be that patients with a PD-1 expression between 1 and 5% behave differently than patients with < 1 and more than 5%. Further studies should do prespecified subgroup analysis for these kinds of patients.

We found significant OS benefit in subgroups male and age greater than 65 years subgroups compared to female and age less than 65 years, respectively, in patients treated with checkpoint inhibitors. Similar to our results, CM 037 trial showed OS was significantly better for age >65-year subgroup compared to < 65-year subgroup. However, OS in KN 006 trial and CM 066 trial did not correlate with different age. Interestingly, there was significantly better in OS CM 067 trial for age < 65-year subgroup compared to > 65-year subgroup. We could not find any possible explanation for this discrepancy. With respect to gender, results of KN 006 were similar to our result. However, OS in CM 037, CM 066, CM067, and CM 069 did not correlate with gender.

Interestingly, we also found significant OS in patients with ECOG 1. However, PFS was significant irrespective of ECOG status, gender, and age. These results can be used to decide treatment of choice and prognostic stratification.

Our meta-analysis has some limitations. First, we did not include individual patient-level data. Second, the results are generalizable only to patient groups eligible for these trials. Significant heterogeneity associated with various outcomes is also an important limitation of our study while interpreting the results. Even though we found significant improvement in overall survival in wild BRAF, PD-1 +, high LDH, Male and age > 65, these outcomes are associated with heterogeneity of 43 %, 40 %, 59 %, 73 %, and 41 %, respectively.

**Conclusion:** Checkpoint inhibitors compared with standard chemotherapy have better survival outcomes in wild BRAF, PD-1 +, high LDH, ECOG 1, Male and age > 65 years subgroups; however, results are weakened by the significant amount of heterogeneity as discussed above. The outcomes of this analysis will assist in the design of future clinical trials based on prespecified subgroups.

## Figures and Tables

**Table 1:** Primary end-point of included studies.

**Study Name (Clinical trials identifier)**	**Study Population**	**Treatment Arms and Dosages**	**Survival Results**
**CheckMate 037** NCT01721746	Advanced melanoma (Stage IIIc and IV) who have progressed on Ipilimumab and BRAF therapy	Arm 1: Nivolumab 3 mg/kg every 2 weeks treated until progression. Arm 2: Investigator choice, which included dacarbazine 1000 mg/m^2^ every 3 weeks or carboplatin area under curve 6 plus paclitaxel 175 mg/m^2^ (Comparison)	**OS** Arm 1: 16 months Arm 2: 14 months HR 0.95 (CI 0.73-1.24) **PFS** Arm 1: 3.1 months Arm 2: 3.7 months HR 1.0 (CI 0.78-1.436)

**CheckMate 066** NCT01721772	Advanced melanoma (stage IIIc and IV) untreated without BRAF Mutation	Arm 1: Nivolumab 3 mg/kg every 2 weeks treated until progression. Arm 2: Investigator choice, which included dacarbazine 1000 mg/m^2^ every 3 weeks or carboplatin area under curve 6 plus paclitaxel 175 mg/m^2^ (Comparison)	**OS at 1 year** Arm 1: 72.9% (CI 65.5-78.9) Arm 2: 42.1% (CI 33-50.9) **PFS** Arm 1: 5.1 months Arm 2: 2.2 months HR 0.43 (CI 0.34-0.56) p<0.001

**CheckMate 067** NCT01844505	Untreated stage III (unresectable) or Stage IV melanoma, With known BRAF V600 Mutation status, And with ECOG of 0 or 1	Arm 1: Nivolumab 1 mg/kg + ipilimumab 3 mg/kg every 3 weeks for four doses followed by nivolumab 3 mg/kg every 2 weeks + placebo Arm 2: Nivolumab 3 mg/kg every 2 weeks + Placebo Arm 3: Ipilimumab 3 mg/kg every 3 weeks + placebo (Comparison)	**OS at 3 years** Arm 1: 58 % Arm 2: 52 % Arm 3: 34% **PFS** Arm 1: 11.5 months HR 0.43 (CI 0.35-0.52) Arm 2: 6.9 months Arm 3: 2.9 months Arm 2 vs 3 HR 0.55 (CI 0.64-0.96)

**CheckMate 069** NCT01927419	Untreated stage III (unresectable) or stage IV melanoma, with BRAF wild type, ECOG of 0 or 1	Arm 1: Nivolumab 1 mg/kg plus ipilimumab 3 mg/kg every 3 weeks for four doses followed by nivolumab 3 mg/kg every 2 weeks plus placebo Arm 2: Ipilimumab 3 mg/kg every 3 week (Comparison)	**OS 2-year follow-up data** Arm 1: 63·8% (CI 53·3–72·6) Arm 2: 53·6% (CI 38·1–66·8) HR 0·74, 95% (CI 0·43–1·26) p=0·26 **PFS at 2 years** Arm 1: Not reached Arm 2: 3 months HR 0.36 (CI 0.22-0.56) p <0.0001

**KEYNOTE 006** NCT01866319	Unresectable Stage III Or IV advanced Melanoma (excluding Ocular melanoma) and up to one previous systemic chemotherapy (excluding anti CTLA- 4, PD-1 or PD-L1)	Arm 1: Pembrolizumab 10 mg/kg every 2 weeks Arm 2: Pembrolizumab 10 mg /kg every 3 weeks Arm 3: IV ipilimumab 3 mg/kg every 3 weeks (Comparison)	**OS** Arm 1: NR (22.1-NR) HR 0.68 (0.53-0.87) p<0.0009 Arm 2: NR (23.5-NR) HR 0.68 (053-0.86) p <0.0008 Arm 3: 16 months (13.5-22) **PFS** Arm 1: 5.6 months HR 0.61 (0.5-075) Arm 2: 4.1 months HR 0.61 (0.5-0.75) Arm 3: 2.8 months HR NA p<0.0001

**KEYNOTE 002** NCT0170428	Unresectable stage III or stage IV melanoma, Confirmed Disease progression on previous BRAF, MEK or CTLA-4 inhibitor therapy. ECOG 0 or 1	Arm 1: 180: Pembrolizumab 2 mg/kg Arm 2: 181: Pembrolizumab 10 mg/kg iv every 3 Weeks Arm 3: Investigator choice chemotherapy (paclitaxel plus carboplatin, paclitaxel, carboplatin, dacarbazine) (Comparison)	**OS: NA** **PFS at 6 months** Arm 1: 34% HR 0.57 (0.45-0.73) p<0.0001 Arm 2: 38% HR 0.5 (0.39-0.64) p <0.0001 Arm 3: 16% in comparison to above arms

**Table 2:** Baseline characteristics of patient demographics.

	**CheckMate 037 Larkin et al. 2017**		**CheckMate 066 Robert et al. 2015**		**CheckMate 067 Wolchok et al. 2017**			**CheckMate 069 Hodi et al. 2016**		**KEYNOTE-002 Ribas et al. 2015**			**KEYNOTE-006 Schachter et al. 2017**		
Phase	**III**		**III**		**III**			**III**		**II**			**III**		

No. of Patients	405		418		945			142		540			834		

Arms	Nivo	ICC	Nivo	Daca	Nivo+Ipi	Nivo	Ipi	Nivo+Ipi	Ipi	Pemb 1	Pemb 2	ICC	Pemb 1	Pemb 2	Ipi

No in arm.	272	133	210	218	314	316	315	95	47	176	177	167	279	277	278

Male	176	85	121	125	NR^1^	NR^1^	NR^1^	63	32	104	109	114	161	174	162

Female	96	48	89	83	NR^1^	NR^1^	NR^1^	32	15	76	72	65	118	103	116

Age in years median	59	62	64	66	NR^1^	NR^1^	NR^1^	64	67	62	60	63	61	63	62

ECOG 0	162	84	148	121	230	237	224	79	37	98	98	99	196	189	188

ECOG 1	110	48	60	84	83	79	91	14	10	80	83	80	83	88	90

**Table 3:** Baseline characteristics of patient molecular characteristics.

	**CheckMate 037 Larkin et al. 2017**		**CheckMate 066 Robert et al. 2015**		**CheckMate 067 Wolchok et al. 2017**			**CheckMate 069 Hodi et al. 2016**		**KEYNOTE-002 Ribas et al. 2015**			**KEYNOTE-006 Schachter et al. 2017**		
Phase	**III**		**III**		**III**			**III**		**II**			**III**		

No. of Patients	405		418		945			142		540			834		

Arms	Nivo	ICC	Nivo	Daca	Nivo+Ipi	Nivo	Ipi	Nivo+Ipi	Ipi	Pemb 1	Pemb 2	ICC	Pemb 1	Pemb 2	Ipi

BRAF Wild	NR	NR	202	204	212	218	215	NR	NR	136	141	138	177	178	170

BRAF Mutated	60	29	NR	NR	102	98	100	22	10	44	40	41	98	97	107

PDL1 Positive	134	67	74	74	*∗∗*	*∗∗*	*∗∗*	24	11	NR	NR	NR	225	221	225

PDL1 Negative	NR	NR	136	134	123	117	113	NR	NR	NR	NR	NR	49	54	47

LDH≤ULN	NR	NR	120	125	199	197	194	70	36	99	105	107	194	175	178

LDH>ULN	139	46	79	74	114	112	115	24	1	77	73	68	81	98	91

*∗∗*Different expression levels are used to stratify, and we have not included data from this study with respect to PD-1 expression outcomes due to non-availability of similar comparison.

Daca = dacarbazine; ECOG = eastern cooperative oncology group; Ipi = ipilimumab; ICC= investigator choice chemotherapy; LDH = lactate dehydrogenase; NR = not reported. Nivo = nivolumab; Pemb1 = pembrolizumab administration every 2 weeks; Pemb2 = q3 administration every 3 weeks; PD= programmed cell death; ULN=upper level of normal. 1 = stratification based on age > 65 years and <65 years.

**Table 4:** Delphi list of included studies.

**Final Delphi List**	**CheckMate 066 Robert et al. 2015**	**CheckMate 067 Wolchok Et al. 2017**	**CheckMate 037 Larkin et al. 2017**	**CheckMate 069 Hodi et al. 2016**	**KEYNOTE-006 Schachter Et al. 2017**	**KEYNOTE-002 Ribas et al. 2015**
**Treatment allocation.** **(a) Was a method Of randomization performed?** **(b) Was the treatment allocation concealed?**	Yes Yes	Yes Yes	Yes Yes	Yes Yes	Yes Yes	Yes Yes

**Were the groups similar at baseline regarding the most Important Prognostic indicators?**	Yes	Yes	Yes	Yes	Yes	Yes

**Were the eligibility criteria specified?**	Yes	Yes	Yes	Yes	Yes	Yes

**Was the outcome assessor blinded?**	Yes	Yes	Yes	Yes	Yes	Yes

**Was the care provider blinded?**	Yes	Yes	Yes	Yes	No	Yes

**Was the patient blinded?**	Yes	Yes	Yes	Yes	No	Yes

**Were point estimates and Measures Of Variability Presented for the Primary outcome measures?**	Yes	Yes	Yes	Yes	Yes	Yes

**Did the analysis include an intention to treat?**	Yes	Yes	Yes	Yes	Yes	Yes

## Supplementary Materials

Supplementary Materials Supplementary Figure 1: funnel plot. Supplementary Figure 2: scatter plot of metaregression using ECOG status. Supplementary Figure 3: scatter plot of metaregression using LDH status. Supplementary Figure 4: scatter plot of metaregression using BRAF mutated status. Supplementary Table1: search strategy for meta-analysis. Supplementary Table 2: list of studies with high risk of bias.
