# Supplementary material for: Clinical and Molecular Characteristics Associated with Survival in Advanced Melanoma Treated with Checkpoint Inhibitors
Source: J Oncol. 2018 Jul 17;2018:6279871. doi: 10.1155/2018/6279871 (PMC6077519; doi:10.1155/2018/6279871)
Supplement: Supplementary Materials — Supplementary Figure 1: funnel plot. Supplementary Figure 2: scatter plot of metaregression using ECOG status. Supplementary Figure 3: scatter plot of metaregression using LDH status. Supplementary Figure 4: scatter plot of metaregression using BRAF mutated status. Supplementary Table1: search strategy for meta-analysis. Supplementary Table 2: list of studies with high risk of bias. [file 6279871.f1.pdf]

Supplementary Figure 1. Funnel plot.

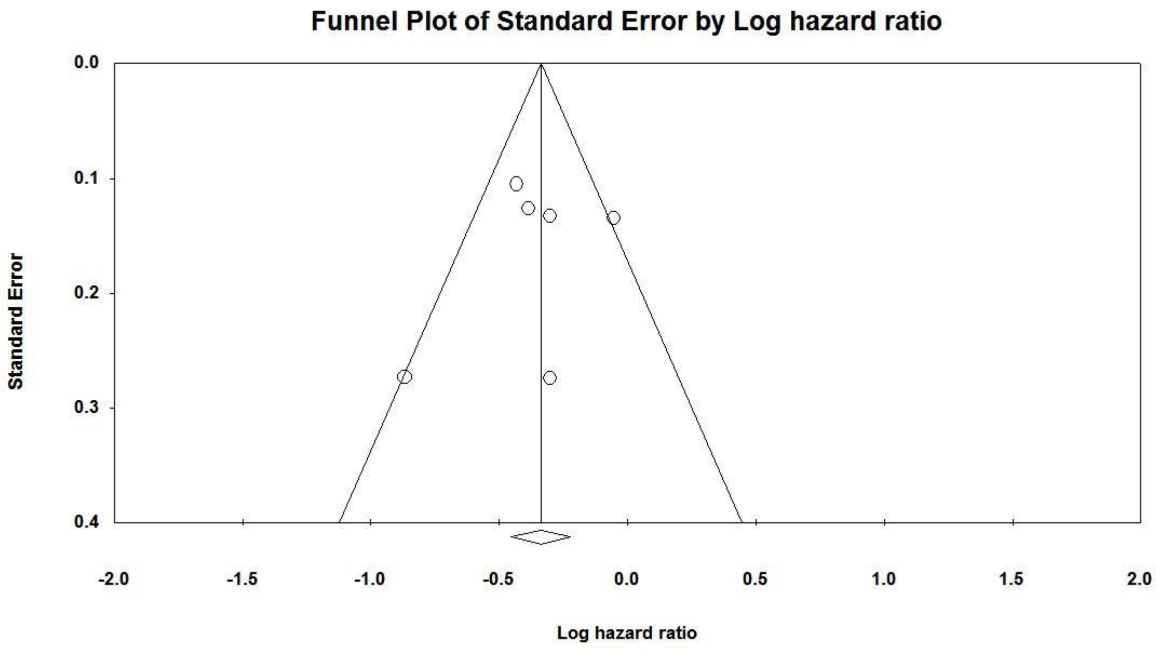

Supplementary Figure 2. Scatter plot of meta-regression using ECOG status.

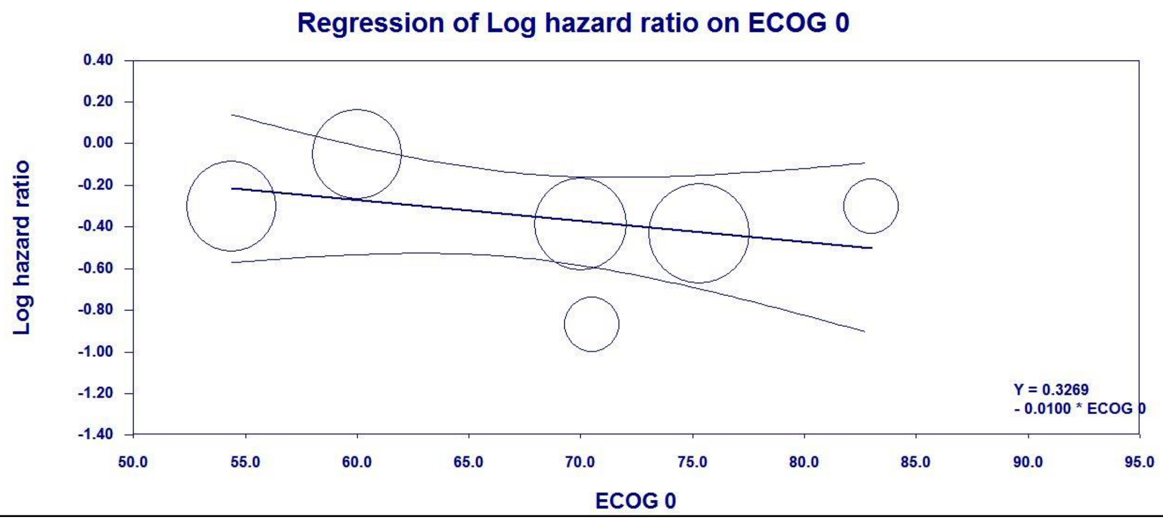

Supplementary Figure 3. Scatter plot of meta-regression using LDH status.

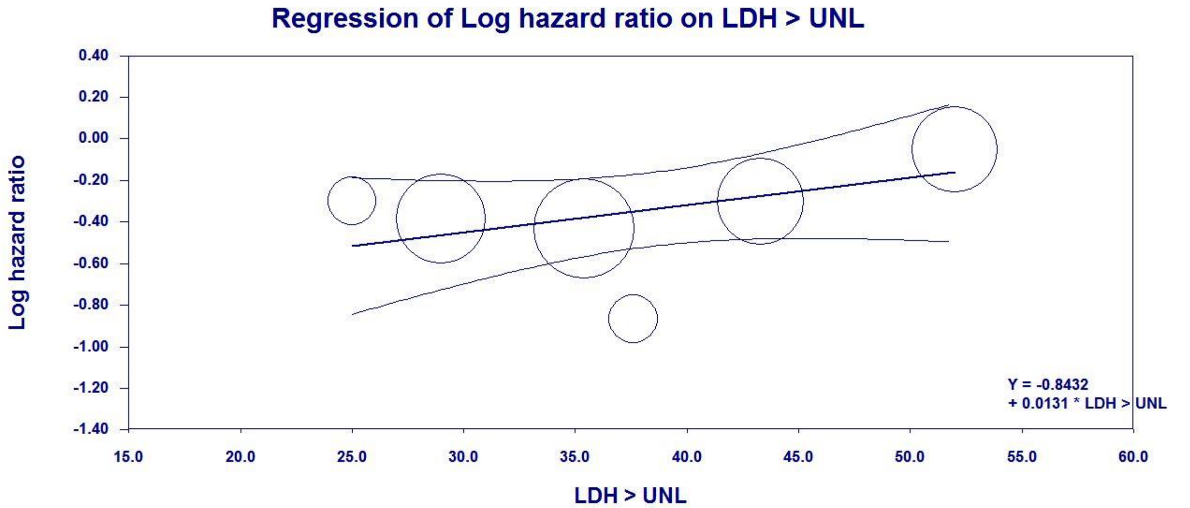

Supplementary Figure 4. Scatter plot of meta-regression using BRAF mutated status.

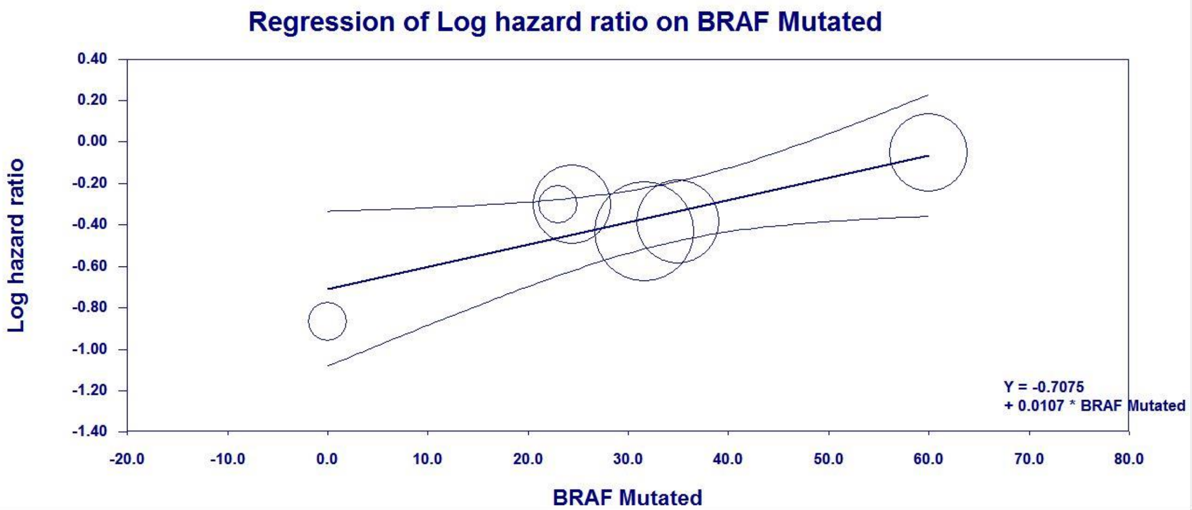

Supplementary Table1. Search Strategy for meta-analysis.

Database	Search Strategy	Number of citations
Cochrane Library	ALL (metastatic AND melanoma OR advance AND melanoma) AND ALL(anti-programmed AND cell AND death AND receptor AND 1 AND monoclonal AND antibody OR anti-PD-1 AND monoclonal AND antibody OR PD-1 OR pembrolizumab OR nivolumab)	128
Scopus	ALL (metastatic melanoma OR advance melanoma) AND ALL(anti-programmed cell death receptor 1 monoclonal antibody OR anti-PD-1 monoclonal antibody OR PD-1 OR pembrolizumab OR nivolumab)	1497
PubMed	#1: all field (metastatic melanoma) OR all field (advance melanoma).<br>#2: all field (anti-programmed cell death receptor 1 monoclonal antibody) OR all field (anti-PD-1 monoclonal antibody) OR all field (PD-1) OR all field (pembrolizumab) OR all field (nivolumab)<br>#3: #1 AND #2	853
Clinicaltrial.gov	Condition or disease: metastatic melanoma OR advance Melanoma<br>Other terms: anti-programmed cell death receptor 1 monoclonal antibody OR anti-PD-1 monoclonal antibody OR PD-1 OR pembrolizumab OR nivolumab<br>Study type: All studies.	150

Supplementary Table 2. List of studies with high risk of Bias.

Study Identification	Reason for possible high risk of bias.
**KEYNOTE 002** NCT0170428	Inherent bias associated with phase II studies including selection and publication bias.
**KEYNOTE 006** NCT01866319	Neither the treating physician nor the patients were blinded leading to high risk for bias.
**CheckMate 066** NCT01721772	Study only included patients with wild BRAF and treatment naïve patients.

List of Abbreviations.

ABBREIVIATION	EXPANSION
BRAF gene	B- Rapidly Accelerated Fibrosarcoma gene
CTLA-4	Cytotoxic T lymphocyte antigen-4
CM	CheckMate
ECOG	Eastern Cooperative Oncology Group
US FDA	United states Food and drug administration
HR	Hazard ratio
KN	KEYNOTE
LDH	Lactate dehydrogenase
MEK	Mitogen-activated kinase
NCCN	National Comprehensive Cancer Network
OS	Overall Survival
PD1	Programed cell death 1
PDL	Programed cell death ligand
PFS	Progression Free Survival
PRISMA	Preferred Reporting Items for Systematic Reviews and Meta-Analyses
PICOS	Population. Intervention. Comparison. Outcome. Study Design
SEER	Surveillance, Epidemiology, and End Results
ULN	Upper level of normal.
